# Self-Medication for Chronic Pain Using Classic Psychedelics: A Qualitative Investigation to Inform Future Research

**DOI:** 10.3389/fpsyt.2021.735427

**Published:** 2021-11-12

**Authors:** Julia Bornemann, James B. Close, Meg J. Spriggs, Robin Carhart-Harris, Leor Roseman

**Affiliations:** ^1^Centre for Psychedelic Research, Division of Brain Sciences, Imperial College London, London, United Kingdom; ^2^Psychedelics Division, Neurology, Psychiatry and Behavioral Sciences Weill Institute for Neurosciences, University of California, San Francisco, San Francisco, CA, United States

**Keywords:** psychedelic, chronic pain, self-medication, Patient and Public Involvement (PPI), thematic analysis

## Abstract

**Background:** Chronic Pain is among the leading causes of disability worldwide with up to 60% of patients suffering from comorbid depression. Psychedelic-assisted therapy has recently been found effective in treating a host of mental health issues including depression and has historically been found to be useful in treating pain. Reports of self-medication for chronic pain using psychedelic drugs have been widely documented, with anecdotal evidence indicating widespread success in a range of pathologies.

**Aims:** In preparation for an upcoming trial, to better understand how those with lived experience of chronic pain self-medicate with psychedelic drugs, and to establish, in detail, their therapeutic protocols and practices for success.

**Methods:** As part of patient-involvement (PI) for an upcoming trial in this population, 11 individuals who reported self-medicating with psychedelic drugs took part in a 1-h semi-structured discussion, which was then transcribed and thematically analyzed.

**Results:** Across a range of psychedelic substances and doses, reported pain scores improved substantially during and after psychedelic experiences. Two processes, Positive Reframing and Somatic Presence, were reliably identified as playing a role in improvements in mental wellbeing, relationship with pain, and physical (dis)comfort. Inclusion of other strategies such as mindfulness, breathwork, and movement were also widely reported. Due to the data's subjective nature, this paper is vulnerable to bias and makes no claims on causality or generalisability. Together, these results have been used to inform study design for a forthcoming trial.

**Conclusion:** This pre-trial PI work gives us confidence to test psychedelic therapy for chronic pain in a forthcoming controlled trial. The results presented here will be instrumental in improving our ability to meet the needs of future study participants.

## Introduction

Public and Patient Involvement (PPI) aims to produce research “by” and “with,” rather than “to” or “for” people with lived experience (NIHR). PPI is effective across health research (1, 2), particularly in mental health research (3, 4), and is an emerging topic in the psychedelic field (5). By incorporating non-researcher, e.g., patient and public input into various parts of the research cycle, PPI produces relevant, transparent, and accountable research (6, 7). This PPI venture was undertaken to involve people with lived experience (“contributor”) in the design phase of a future trial investigating the effects of psilocybin in people with fibromyalgia. The fibromyalgia population was chosen for potential psychedelic application because of its relatively common occurrence, central nervous system involvement, and high rates of mental health co-morbidities.

Those living with chronic pain report feeling misunderstood and invalidated (8, 9). This highlights the need for intentional communication at early stages of research to ensure thoughtful design that prioritizes the specific needs of people with chronic pain. The accordingly selected methods of in-depth, open-ended discussions (10, 11) and the thematic analysis thereof (1, 12–14) are widely used in PPI. Further, such conversations effectively facilitate early involvement at the design stage of research (10, 15). The product is a nuanced exploration of the chronic pain experience and warrants the following background to adequately contextualize.

Roughly 20% of the global population live with chronic pain (16), and it is considered one of the global leading causes of disability (17). Chronic pain is defined as pain lasting over three months and may remain even if the original injury has formally healed (18, 19). Common chronic pain conditions include Chronic Low Back Pain, Headache, and Chronic Widespread Pain e.g. Fibromyalgia Syndrome. Living with chronic pain significantly impacts a person's ability to work, resulting in high levels of lost productivity, reportedly costing the UK economy £10.7 billion annually (20). The social implications of chronic pain are also considerable; over half of pain patients report that their condition has prevented them from seeing family and friends and that their pain contributes to significant social anxiety (21). Such increases in social isolation, as well as the general stress from constant pain, directly impact patients' psychological wellbeing; it is estimated that 20% of people with chronic pain experience comorbid depression (22), with rates up to 60% in conditions such as Fibromyalgia Syndrome (23). This well observed bidirectional relationship links stress to an increased likelihood of developing chronic pain (24, 25).

Current guidelines recommend education, physical therapy and pharmacological interventions, such as non-steroidal anti-inflammatory drugs (NSAIDs), weak opioids, and antidepressants or anticonvulsants off-label (26, 27). Second line treatments graduate to invasive procedures such as neurosurgery, neuromodulation, nerve blocks and radiofrequency denervation (27, 28). However, several problems with the current strategies for treatment of chronic pain remain. Firstly, existing medications carry a number of unwanted side effects and can be habit forming, for example long-term opioid use is linked to dependence and has directly contributed to the ongoing international opioid crisis (29). Secondly, conventional interventions, both pharmacological and invasive, have high Number-Needed-to-Treat values and are only effective in up to 80%, leaving ~1 in 5 sufferers without pain relief (30). Finally, these treatments either neglect or fail to adequately address the psychological impact of chronic pain conditions, leading to growing numbers seeking alternative treatments and experiment with self-treatment (31, 32). In cases where pain persists, patients can be referred to multidisciplinary pain management programmes (PMPs), which aim to support individuals to effectively manage and live with their pain through education, physical therapy and psychological therapies such as Cognitive Behavioral Therapy (CBT) (33) or Acceptance and Commitment Therapy (ACT) (34). CBT and ACT are well-established trans-diagnostic behavior-based psychological therapies which aim to improve awareness and reduce harm caused by negative thought patterns. Their shared foundation focuses upon Cognitive Reframing/Restructuring, a process in which thoughts and beliefs are identified, examined, their relative importance is reviewed, and maladaptive beliefs are updated with therapeutically useful, often positive, ones (35). Evidence suggests such cognitive reframing is transdiagnostically useful, resulting in improved outcomes in healthy populations (36), depression (37), Post-Traumatic Stress Disorder (PTSD) (35), and chronic pain (38). While PMPs are the sole treatment option to directly address the prevalent mental health comorbidities associated with chronic pain, their efficacy is largely limited to the short term (39–42) and attrition/relapse rates remain high (43, 44).

Used in combination with psychological support, psychedelic drugs [e.g., Lysergic Acid Diethylamide (LSD), Psilocybin (the psychoactive component of magic mushrooms), and dimethyltryptamine (DMT)] appear to exhibit promising therapeutic effects in conditions such as depression (45–47), addiction (48, 49), and end-of-life anxiety (50–53). The safety profile of psychedelics is well-established as largely physiologically benign (54), though psychologically challenging periods are common during acute experiences (55). Case reports of persisting perceptual changes exist, though these are rare (54, 55). Historically, there has been interest in using psychedelics to treat chronic pain; preliminary studies from the 1960s and 70s suggest that psychedelic drugs may be therapeutically useful, specifically for cancer pain and phantom limb pain (56–61). Although the results of these historical trials all show promising results, they lacked the methodological rigor of modern trials making it difficult to draw strong inferences on their findings. Contemporary studies suggest that psychedelics may be therapeutically useful in treating intractable headaches such as migraine and cluster headaches (62–64), and two recent reviews hypothesize potential mechanisms and applications for psychedelics in chronic pain (65, 66). Pharmacologically, this concept is plausible. The primary mechanism of action of classic psychedelics is *via* the 5-HT_2A_ serotonin receptor, which is integral to inflammatory pain (67, 68). Data suggests that psychedelics reduce inflammation (69–71) *via* the downstream effects of 5-HT2_A_ agonism such as TNF regulation (72), and may result in desensitized central pain responses (66). The acute effects of psychedelics may also contribute toward an analgesic response by reorienting attention away from unpleasant sensations toward altered perceptions, e.g., visual hallucinations (73).

While research has stalled for decades due to the legal status of psychedelic drugs, public interest has not. Psychedelic self-medication has grown in popularity in recent years, reportedly making up 14.8% of self-reported psychedelic substance use (74) and first time LSD-use having increased ten-fold in 10 years in adults over 26 (75). Methods of use range from the semi-regular taking of sub-perceptible doses (known as “microdosing”) to isolated high dose sessions emulating clinical contexts to address mental health concerns (76). Notably, anecdotal reports of effective management of chronic pain have been prevalent; online forums such as Erowid and Reddit contain hundreds of reports of effective treatment for chronic pain conditions such as Fibromyalgia (ErowidFMS) (77, 78), Chronic Back Pain (ErowidCBP), and Rheumatoid pain (RedditEDS) (79). The largest group of reports concern chronic cluster headaches and migraines (80), with over 10,000 people participating in the organization “Clusterbusters,” dedicated to the treatment of headaches with psychedelics (81). This has given rise to a cache of largely untapped knowledge held by psychedelic users who, through self-experimentation and extensive online publishing of “trip reports,” have independently attempted to develop a public library detailing the effects of different substances. This PPI initiative takes advantage of existing public interest and knowledge with the objective of infusing context and input from people with lived experience into various aspects of trial development, and directly influence study design, therapeutic protocols, and outcome measures.

For this PPI investigation, 11 public contributors were invited for 1-to-1 open ended discussions with one of the researchers developing the upcoming trial. The aim of these discussions was to explore (i) how people with chronic pain self-medicate with psychedelic drugs, (ii) whether people have found psychedelics to be effective, and (iii) to gain some insight on specific practices that contributors feel are important for treatment success. While safeguards against bias were employed (see section Materials and Methods), we do not claim that the following work is free of bias or generalisable to the larger population.

## Materials and Methods

### Public Contributors

Contributors with lived experience of chronic pain and alleged personal use of psychedelics as a self-medication attempt were recruited through online pain forums, e.g., Reddit, psychedelic retreats, and word-of-mouth. Online posts asked about experiences with psychedelic self-medication for chronic pain and whether people would be open to a discussion about this topic. In total, 44 people responded, and 11 agreed to a 1-h video conversation. The remaining 33 respondents did not contribute to this project because they either failed to reply to messages requesting a conversation, or did not appear at the agreed-upon meetings (3). Eight contributors were recruited from online forums, one was recruited through word-of-mouth, and two were recruited from psychedelic retreats *via* advertisements sent to retreat alumni email lists.

### Discussions

Discussions took place between April and June of 2020 and lasted between 55 and 90 min. Consent was given to record, analyse, and use data obtained from discussions in analysis. Discussions were semi-structured and spanned three major topics: a background of their pain, their psychedelic use, and whether and how psychedelic use may have been effective for their chronic pain. This open-ended approach was chosen to capture a range of responses and reduce interviewer bias. All contributors were asked to rate their pain at various timepoints according to a Numerical Pain Rating Scale (NPRS) from 0 (No Pain) to 10 (Worst Imaginable Pain) (82). The common interpretation of this rating scale is that pain scores of 1–3 are considered to be mild, 4–6 moderate, and 7–10 severe. If contributors reported multiple experiences, their most impactful experience was selected. Contributors were asked to rate the pain that they experience (1) on an everyday basis, before they began self-medicating with psychedelics, (2) their pain during the acute experience, and (3) after they self-medicated with psychedelics (see Figure 2). Discussions were recorded and transcribed.

### Ethical Approval

The Imperial Research Governance and Integrity Team (RGIT) was informed of all planned activities of the following project involving human PPI contributors. This project was undertaken as PPI and RGIT confirmed that ethical approval was not required. During conversations, PPI contributors provided verbal informed consent to participate in this study.

### Data Analysis

We used qualitative methods to derive major trends across contributors. To obtain an unbiased view of the data, thematic analysis followed an inductive coding approach (83), allowing themes to arise from the transcriptions themselves, and minimizing confirmation bias. Analysis was undertaken on each data set and created initial codes. Once a first set of codes was established, the sample set was re-analyzed, updated codes were produced, and themes were determined. Subjectivity was addressed by cross-referencing for inter-coder reliability and the final set of themes was confirmed. From this, the themes were categorized into Pain Disability/Debilitation, Acute, and Enduring Change categories and configured into a hierarchical coding frame to establish the relative importance of each theme. This produced major and minor themes. Themes were considered major if they appeared in at least four discussions. There were several major themes which contained several sub-themes.

**Figure 1:**
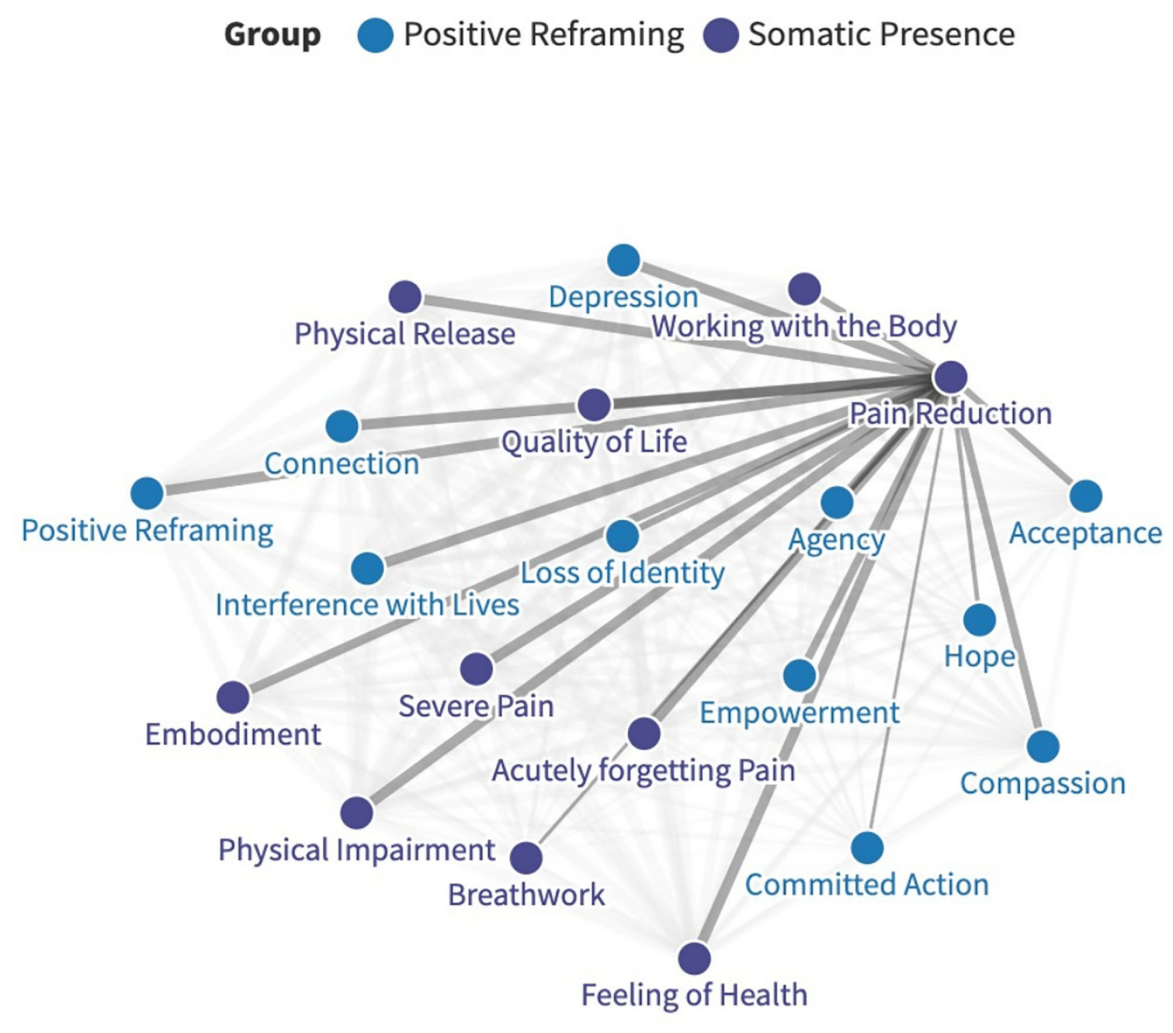
An example of how relative connection strengths were mapped on a network graph, in this case regarding pain reduction. The full, interactive graph is available here: https://public.flourish.studio/visualization/6283835/.

To establish the relationships between themes, one matrix was created for all contributors and themes, and the presence of each theme was detailed. The relative strength of connection between themes was established through the co-occurrence of themes, which were coded by frequency. These connections were noted and mapped onto a network graph using open access software (Figure 1) (84). No quantitative, statistical analyses were undertaken as the values reported were retrospective, and the main aim was to explore relationships between themes. Therefore, the following serves as a commentary on how themes were interrelated and makes no inferences about causation.

**Figure 2:**
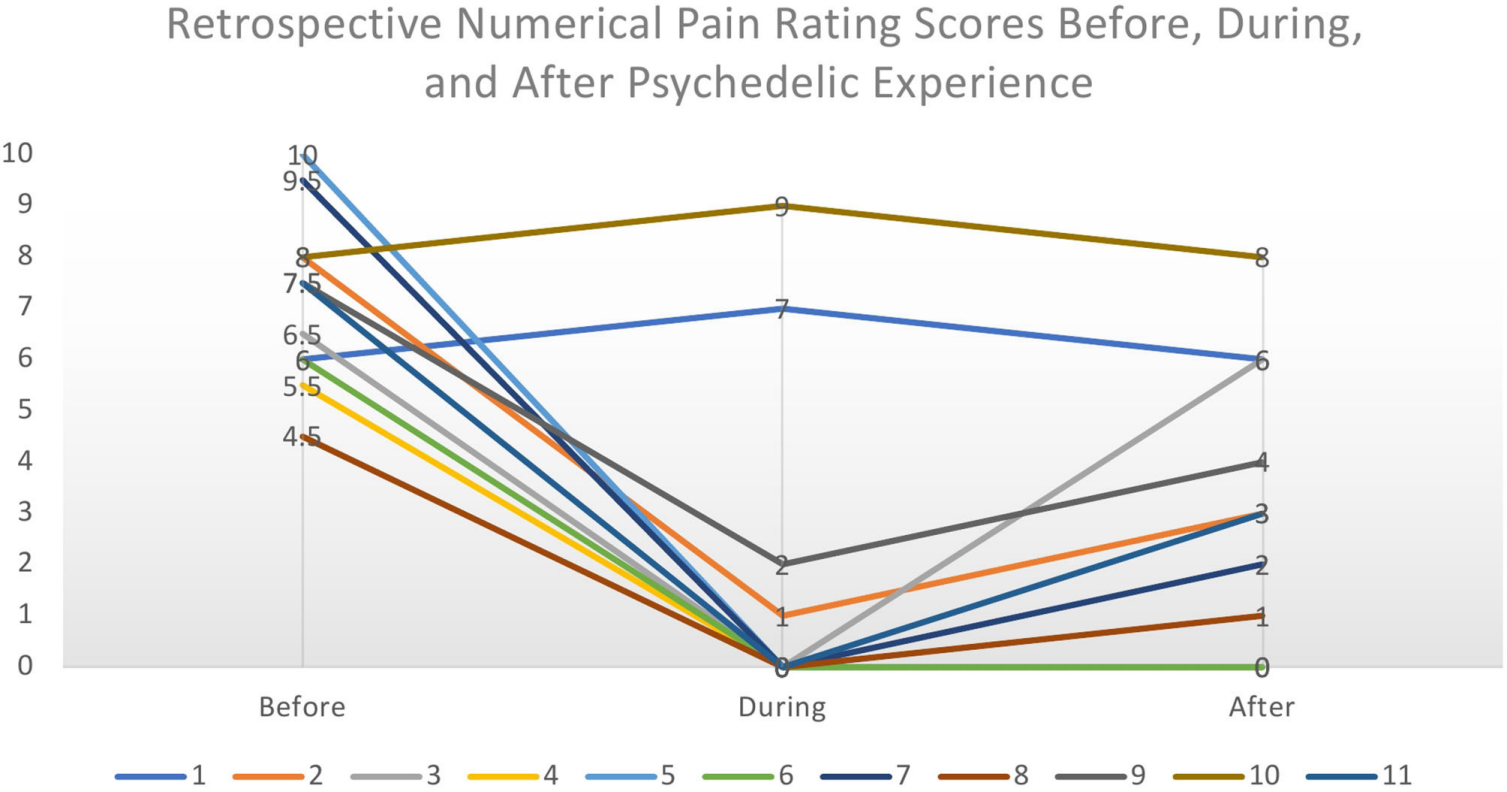
Retrospective Numerical Pain Rating Scores from each contributor before, during, and after the psychedelic experience. The perceived intensity of pain was considerably lowered during the acute psychedelic state and slowly increased to the normal level over a period of hours to days. We make no inferences on causality from these retrospective subjective data.

### Reporting

This investigation followed GRIPP2 guidelines for reporting PPI (85). PPI was undertaken at the planning stage to influence the design of our study. Contributors were not paid for their involvement.

## Results

Demographics of the six women and five men (range = 21 and 52, mean = 34, SD = 9.7) years of age) who took part in these conversations can be found in Table 1. Ethnicities were slightly over-represented when compared to the UK national population. Contributors had mixed chronic pain, six of which reported more than one contributing condition. Contributors had been living with their pain for between two and 25 years (mean = 10.7, SD = 9) and tried between two and 14 pain treatments (mean = 6.6, SD = 4.4) with largely unsatisfactory rates of success. All contributors had previously received pharmacological treatment, and three pursued more invasive treatment such as radiofrequency ablation and surgery. All contributors reported co-morbid depression that was perceived to be closely linked to the intensity of their pain. Histories of psychological treatments were not provided.

**Table 1:** Summary of contributor demographics.

	**Frequency (*n*)**	**%**
**Sex**		
Female	6	55%
Male	5	45%
**Ethnicity**		
White	7	64%
Black	2	18%
Hispanic	1	9%
Mixed Race	1	9%
**Employment status**		
Employed Full-Time	5	45%
Unemployed	2	18%
Student	3	27%
Unable to work	2	18%
**Nature of pain**		
Traumatic injury, e.g., whiplash	6	55%
Congenital condition, e.g., Femoral acetabular impingement	3	27%
Connective tissue condition, e.g., Ehler's Danlos syndrome	3	27%
Chronic back pain	3	27%
Fibromyalgia syndrome	2	18%
Autoimmune disorders, e.g., Hashimoto's disease	2	18%
**Past prescribed treatments**		
Opioids, e.g., Hydromorphone	6	55%
Anti-inflammatory agents, e.g., Meloxicam	7	64%
Neuropathic agents, e.g., Gabapentin	4	36%
Ketamine	4	36%
Muscle relaxants	2	18%
Surgery	2	18%
Radiofrequency ablation	1	9%
**Past self-directed treatments**		
Cannabis	6	55%
Kratom	2	18%
Acupressure	2	18%

### Substances, Doses, and Frequency-of-Use

There was considerable variation in substances, doses, frequency-of-use, and the longevity of effects reported (see Table 2). This is likely due to the lack of research on the subject, as well as the absence of any guidelines due to legal status, leading contributors to experiment and create unique “treatment plans.” These plans were largely the product of trial-and-error, especially in regard to substance and dose.

**Table 2:** Summary of substances, dose, supplementary treatments, and pain responses.

	**Frequency**	**%**
**Substance**		
LSD	5	45%
Psilocybin-containing mushrooms	9	82%
Psilocybin-containing truffles	2	18%
DMT	3	27%
Ayahuasca	1	9%
**Dose**		
Microdose (≤ 20 μg LSD, ≤ 400mg psilocybin)	5	45%
“Full” dose	11	100%
**Supplementary treatment**		
Breathwork	4	36%
Meditation and mindfulness	7	64%
Movement, e.g., Yoga, Qi Gong, and physical therapy exercises	5	45%
Expressive outlets, e.g., art, journaling, dance	4	36%
**Acute pain response**		
Complete analgesia	9	82%
Partial analgesia	5	45%
Pain amplification	3	27%
**Lifetime use**		
Once	1	9%
2–10	3	27%
11–20	2	18%
21–50	4	36%
50+	1	9%

As shown in Table 2, the substances used ranged, the most reported being psilocybin-containing mushrooms. Although not a serotonergic psychedelic and therefore not included in the analysis of this project, three people also reported using ketamine. There was also a wide range in precision, dose, frequency, and lifetime use; all reported taking at least one “high” dose of psilocybin, the estimated mean of which was 2.6g (SD = 0.5). High doses were largely taken on an as-needed basis, typically separating doses by several months. Some (5) also reported microdosing, on average twice weekly, with psilocybin (*n* = 3; mean dose = 225 mg; SD = 127.5) and LSD (*n* = 2; mean dose = 10 μg; SD = 5). Lifetime use ranged from 1 to >50 experiences.

### Pain and Effect of Self-Medication on Pain

Retrospective baseline pain severity (NPRS) scores ranged from 4.5 to 10/10 with a mean score of 7.25/10, implying severe chronic pain. All contributors reported a change in pain scores during the acute experience; nine reported pronounced reductions while two reported a short-term amplification of pain. Of those who reported a reduction in pain, the mean was 0.33/10. Finally, contributors were asked to rate their everyday pain after starting to self-medicate with psychedelics, focusing on the immediate period *after* the acute psychedelic effects had subsided, i.e., the following 48 h. Scores ranged from 0 to 8, with a mean of 2.73/10, implying mild pain. For most (7), this was followed by a gradual return toward baseline pain scores over a 2–5 days. The two who reported an increase during the acute experience reported a return to baseline pain scores following the experience. Given the exploratory nature of these data, it is not possible to determine the impact of different substances or dosing regimens to the changes in pain score.

The period of direct pain relief ranged from none (2) to 6+ months (2) (mean = 36, median = 7 days, SD = 67.9). Following thematic analysis, effects were split into somatic, i.e., analgesic, and psychological relief. In most cases, perceived longevity of “analgesia-like” pain relief was much more homogenous and ranged from 3 to 7 (mean = 5.4, median = 7, and SD = 2.9) days. Psychological effects were longer lasting, with four contributors reporting changes lasting for several months. One contributor was “not certain” of longevity, and six contributors reported that psychological changes had endured indefinitely after beginning their self-medication. Most (8) contributors spontaneously reported that they hoped that psychedelic-assisted therapy be included in the future canon of pain treatments.

### Thematic Analysis

Thematic Analysis revealed three major categories of themes relating to Pain Disability/Debilitation, Acute experience, and Enduring changes. Each category contained major and minor themes. Larger major themes were split into several sub-themes. Themes are reported by major category of change, either “Positive Reframing,” or “Somatic Presence.” The final categories and contained themes and sub-themes are outlined below (Table 3). Interactive network graphs detail the relationships between all major themes are available here: https://public.flourish.studio/visualization/6283835/ (see Figure 1). Themes are described below with quotes from discussions:

1. Positive Reframing: This category describes contributors' psychological journey from depression, hopelessness, and pain catastrophising toward subjective experiences of acceptance and empowerment. Shifts toward perceived optimism and mental wellbeing were reported after psychedelic use and contributors described viewing often-unchanged situations, e.g., their life and pain, from hopeful and compassionate perspectives.
1.1 Pre-dose Impact of Pain: This category encompasses themes that contributed toward the burden caused by pain before psychedelic self-medication and provides context for the changes observed afterwards. While the physical and mental aspects of this category are invariably intertwined, this section will focus on the latter only. The quality of life of contributors was severely compromised by pain-related pessimism which affected their motivation and perceived ability to regain health. The following quotes suggest that the psychological strain of chronic pain was at least as impactful as the pain itself.
1.1.1 Depression: All contributors reported feelings of depression as part of their everyday life. While some contributors reported feelings of depression preceding their pain (5), all reported that their mental wellbeing and subsequent depression were impacted by the chronification of their pain. Several contributors (3) reported feeling suicidal due to their pain. Contributors reported feelings of hopelessness (7), frustration (7), distress (4), anxiety (9), defeat (4), anger (7), pain feeling all-encompassing (6), and lack of control (9).

“*It's easy to feel super defeated when you can't do anything… It feels like my life is a candle and I'm just watching the best part of my life burn away in front of my face and I can't really do anything about it*”—*C7*.

“*[After diagnosis, I thought] I'm going to die a painful death and my life is going to suck. Woe is me, I'm not going to make it to 60… I was overloaded with sadness*”—*C3*.

“*I just remember feeling really frustrated that I was in this situation*. ‘*When will it end, when will I feel normal again?*'…* I felt like I was my pain was controlling my life. There's some hopelessness in that… (I) legitimately thought I was probably never going to get better, or that I would never feel true happiness again, or comfort, or any of those things*”—*C11*.

1.1.2 Interference with Lives: Pain interfered with the progression of the lives of all contributors, in the realms of career (7), relationships (3), and mood (10). This contributed to perceptions of hopelessness and a lack of control described above.
1.1.2.1 Psychological Disposition. Ten contributors reported a link between their pain intensity and their psychological disposition and mood. Of these, seven reported that specifically stress, both chronic and acute, contributed to pain severity. Acute stress worsened pain for six contributors and contributed to feelings of overwhelm. Five contributors reported that they felt chronic stress contributed to the development of their pain.

“*When I've had breakthroughs in my therapy, the pain has gone down. That's probably the biggest correlation between my trauma and my pain*”—*C1*.

“*There was, and still is, a very clear correlation between my stress and emotional state and my level of pain*”—*C11*.

“*I notice (the pain) more in the times when I'm really stressed out*”—*C4*.

1.1.3 Loss of Identity: Seven contributors reported, to varying extents, feeling as though they lost part of their identity due to their pain. Contributors reported a change in personality due to medication (2), feelings of dissociation (4), and inability to perform activities, e.g., sports that were previously integral to their life and identity (3).

“*(Diagnosis) was really challenging. I worked closely with my therapist, and it was like navigating the different stages of grief… When I was diagnosed, to me it felt initially like my whole life was over. I didn't know what my identity was anymore…I wasn't sure if I was going to be wheelchair bound, if I was going to lose the use of my legs, what my life was going to look like*”—*C3*.

1.1.4 Desire for Escape: Three contributors reported feeling a desire to escape from their pain and their body, ranging from desperation for relief to dissociation.

“*I just want a break; I just want to be happy. I'm so depressed. I don't want to be like this. How do I get out of this?*”—*C3*.

1.1.5 Self-Punishing: One contributor used pain as a form of self-punishing by intentionally exposing themselves to known triggers.

“*If I feel like I haven't been my best self, if I feel like I'm not following my truth on some level. I think it can flare up in that context as a reminder, as a punisher in a way*”—*C5*.

1.2 Post-dose Enduring Change: All contributors reported enduring changes in perspective, specifically positively reframing their relationship to themselves and their pain. Agency, acceptance, hope, and confidence were recurring factors in improved pain management and general wellbeing.
1.2.1 Mental Wellbeing: All contributors reported an increase in mental wellbeing. Consistent with literature (47), they particularly reported antidepressant and anxiolytic effects.

“*I legitimately considered killing myself a few weeks ago. I feel like my life has been saved… I have more of a will to exist now*”—*C7*.

“*Every time I've done (mushrooms), it's always reminded me that there's a light at the end of the tunnel, that (the pain) might suck, but it's not that bad. It's totally manageable, I can be happy while all of this is going on*”—*C3*.

“*Trying to focus on the positive and being grateful and being aware of all the wonderful things that are going on in their life. That really does make a huge difference for me*”—*C2*.

1.2.2 Acceptance of Pain: Seven contributors reported increased acceptance of their situation and found this to be helpful in their general experience of pain.

“*I do feel differently about it. I also think differently about it… I'm able to say, yes, (pain) is something that I deal with. And yes, it sucks. And yes, I don't have the resource to do anything about it right now, but I'm doing what I can, and I'm still here, and I'm still alive. And it's not really that bad. I'm able to look at it differently, and that has a big effect*”—*C4*.

1.2.3 Increased A*g*ency: Seven contributors reported feeling more in control over their pain and life.

“*I still experience pain, but I experience it differently… Since I started microdosing mushrooms, I feel like it almost slows me down. I still have the same life stimuli coming at me, that hasn't changed, but it's allowed me to slow down and analyse the stimuli and then really think about how I want to react to it. That's the same with my pain. When I have a flare up of my pain, I really do feel like it's allowed me to have a little more time, or maybe thought space to process. I think, “Okay, you're hurting. What are the reasons? Is it because you did too much exercise or you sat down too long or is it emotional or stress based? How are we going to deal with this?*”—*C11*.

1.2.4 Committed Action: Four contributors reported feeling increased motivation and dedication to take care of themselves and their health.

“*That experience inspired me to do things that would make me feel better and in turn feel less pain*”—*C6*.

“*There are things in life that are also contributing to your experience of life. If you are dealing with issues that add to your emotional pain, those things don't lead you to go to the right state of mind… I use the psychedelic experience to cultivate more of the thoughts and feelings that I want in my life… If you are in the right state of mind, you do the things that help with your pain, like eating well, like practicing yoga every day. It impacts a lot of things in your life that will help in the long run with your battle with pain*”—*C8*.

1.2.5 Increased Compassion: Eight contributors felt more compassion toward themselves (6) and others (5).

“*I think the biggest take-away for me was the knowing that I am good… I think that insight turned into compassion for myself* ”—*C9*.

“*Now we've had this huge stress with COVID, I think I've been able to cope a great deal better. Having started the year with the psychedelic experience makes you incredibly more compassionate and connected to others, which is a huge resource in a time like this to tap into*”—*C5*.

1.2.6 Increased Hope: Four contributors reported feeling more hopeful.

“*For me, it was really about hope. Trying to trust that what my brain was telling me wasn't permanent—the depressive thoughts, the awful feelings, how uncomfortable I am. There are ways for my brain to decipher that in a different way. The psychedelics are the hope of that things might be different, they might be better, and my brain might not interpret such awful discomfort. It's that moment of hope, of thinking I might be able to get my abilities back*”—*C3*.

1.2.7 Connection: Psychedelically induced connectedness has been described extensively in existing literature as an essential mediator of wellbeing (86, 87). All contributors claimed feeling more connected, particularly to themselves. Many also felt connected to others (8), nature (4), and spirituality (4). All feelings of connections began in the acute experience and endured beyond.
1.2.7.1 Self: All contributors claimed feeling more connected to their sense of self. This included connection to emotions (4), intuition (3), inner child (1), a sense of identity (5). This self-connectedness contributed toward overall reframing by nurturing perceived self-belief and empowerment (see above), which improved their ability to manage their pain.

“*The experience allowed me to sure up a little bit my own personal view of myself... The truth rained down that I'm a good person. I just wasn't convinced of that before… I was able to undo that sense of being a curse*”—*C5*.

“*(Psychedelics) always change my perception of myself. And my self-esteem. Always. I don't think it's inflated, but it goes to normal*”—*C9*.

2 Somatic Presence: This category explores contributors' physical experiences, and mindfulness thereof. There emerged a repeated narrative from disability toward reduced pain and increased function. The process remains unclear but acute reports of perceptions of both embodiment and physical catharsis are repeatedly implied as possible contributors to change.
2.1 Pain Disability: This category describes the severity of day-to-day impairment contributors experienced due to their pain and serves as a counterpart to the previously described psychological measures. Again, this section provides a useful background for the striking outcomes reported.
2.1.1 Severe Pain: All contributors experienced severe day-to-day pain which felt “overwhelming” and generally remained “no matter what” contributors did.

“*It feels like someone is holding (a branding iron) on my body for the duration of my life*”—*C7*.

“*It's the kind of pain where you're rocking back and forth, unable to handle it… (It) feels like my body is on fire...It feels like every cell is burning, but at the same time is dehydrated and going through radiation... It kind of feels like a sunburn, but not just on the skin level*”—*C10*.

2.1.2 Physical Impairment: All contributors claimed physical impairment in the form of general functioning (11), sleep (7), or through the failure of previous treatments (7). Contributors claimed feeling disembodied and exhausted by their pain, both physically and emotionally, and felt “desperate” for relief.
2.1.2.1 Sleep: Seven contributors reported their pain affecting their sleep with severity ranging from difficulty sleeping (5) to not sleeping for several days (2). Beyond sleep, most contributors (10) reported feeling fatigued from the pain.

“*I was going for days without really sleeping and trying to find a comfortable way to sleep”—C11*.

“*I couldn't even sleep, I literally could not sleep…I could handle the pain for the most part. It's just pain. But when I can't sleep at all, that's just something else entirely*”—*C7*.

“*The fatigue was to a point where I needed help getting out of bed*”—*C10*.

2.1.2.2 Function: General functioning was impaired for all contributors. This encompassed several realms, including eating (2), ability to do household work (3), moving around (7), getting easily triggered from the environment (1), memory loss (3), developing tics as coping mechanisms (2), and loss of independence (2).

“*I didn't cook dinner for my children for over a year. I couldn't run errands or drive. The pain is debilitating*”—*C11*.

“*I can't walk and can't move... It gets to the point where I can't move an inch… it just feels like I'm on fire. It just won't stop no matter what I do. Recently, even just laying there hurts, everything hurts: walking, going to the bathroom… I can't even go to the grocery store*”—*C7*.

2.1.2.3 Failure of Previous Treatments: For many contributors (7), treatment added considerable strain to their lives. Out of six contributors who had previously been prescribed opioid medication, two reported fear of treatment due to addiction potential and therefore abstained, and two reported developing an opioid dependency which had serious negative impact on their life. For other medications, contributors reported medication impacting cognition (1), worsening symptoms (4), and ability to stay awake (1). Five contributors reported medication either never working (3), or reducing in efficacy over time (2), effectively leaving them without options. Five contributors either underwent (2), or were expecting (3) surgery, with one contributor reporting considerable side effects thereafter.

“*I think over time my pain increased with the more opioids I took… I had no idea that I had become dependent... After taking myself off opioids and making it through the withdrawal symptoms... I was suffering from acute depression and anxiety in addition to my pain condition It spiraled me into a depressive state…I feel like (opioids) fucked with my head, made me a different person*”—*C11*.

“*(On Prednisone) I was literally screaming myself into headaches and grinding my teeth smooth all night for weeks trying to sleep and (it) did not help (the pain) at all. Life was getting worse every day*”—*C7*.

“*(Gabapentin and Lyrica) make me flighty, and I forget things—that's not my personality at all… (They) made my mind go. It was like I was a dementia patient*”—*C3*.

2.2 Acute: During the psychedelic experience itself, contributors reported changed perceptions of pain mostly through analgesia (9), though two contributors reported subjective pain amplification. The process by which this occurred is not clear, though both active and passive processes were implicated in outcome. Varying degrees of physical catharsis (11) were observed, which were often (8) induced or enhanced through intentional focus on the body and breath.
2.2.1 Acutely Forgetting Pain: Most contributors (9) reported reduced pain during the psychedelic experience that took form as complete (9) or partial (5) analgesia.

“*(Acutely,) I don't think of my pain any longer...I don't focus into pain because it's not there*”—*C8*.

“*I felt zero discomfort in my body. I really didn't even think about my pain throughout my experience, which was really remarkable*”—*C11*.

“*I remember getting up… and just being absolutely painless… I was standing up, perfectly upright, straight. Normally I can't put any pressure whatsoever on the right side of my body… I use crutches and canes most of the time, but I haven't for a few days*”—*C7*.

2.2.2 Feeling of Health: Nine contributors reported a restored perception of health in their body. While descriptions largely related to the body, 10 contributors also claimed to feel mentally healthy. As with previous themes, the distinction is not clear as many contributors viewed health as including both body and mind (a continuous theme—see “Embodiment”).

“*My body felt amazing. It felt normal, it felt healthy… Like taking a deep breath and just letting it out and this full body feeling of euphoria*”—*C3*.

“*My back was starting to feel like my other muscles, which is a huge difference for me…That's like when I'm on DMT—it feels normal, which is such a foreign word to my mouth to just feel normal*”—*C4*.

2.2.3 Pain Amplification: Pain was amplified acutely for three contributors who all found the experience to be distressing. Contributors reported increases in pain and sensitivity at moderate and high doses (2), benefitting in spite of the pain (2), and that this amplification was only experienced at an extremely high dose and was contrary to previous experiences (1).

“*I was focused on my pain and I realized that there's nothing I can do. I was going in there deeper and deeper, and I was just feeling it more and more and it was really painful but I couldn't actually get to the source of it for some reason. So that's when I realized that* ‘*okay, this is serious, I have to seek help*.' *That was my actual reason for seeking help*”—*C1*.

2.2.4 Physical Release: All contributors reported some form of perceived physical release through crying (3), tingling (5), vocalization (2), change in temperature (1), and release of muscular tension (5).

“*I noticed that while on mushrooms, I could actively feel where there's tension in the body. In yoga, or any practice for healing, they talk about breathing into that space, relaxing that space. I felt I was actually able to do that on the mushrooms*”—*C9*.

2.2.4.1 Somatic Discharge: More violent expressions of physical release were considered as somatic discharge (2), defined as the process by which trapped emotions are discharged from the body in a cathartic expulsion (88).

“*I felt this intense welling of energy starting from the core of my body and it started to overtake me… (I) started crying, breaking down, just letting it all go, howling in agony and all this suffering I was just letting it out… I allowed it to take over, my body was moving, writhing and squirming… My entire body was tingling and felt really light and I felt so relaxed and completely pain free… It was the biggest release of my life… I got to let go of so much grief, fear, and even some anger. It*'*s like every single cell in my body is just experiencing bliss, all at once. I really felt healed*”—*C6*.

2.2.5 Working with the body: Eight contributors reported actively working with their body through focused attention (4), breathing (4), visualization (5), and movement (4).

“*I was laying on the floor and just breathing in different spots and feeling the energy in my body. I had more access to breath and (was) able to breathe into the spot and to actively feel the muscle, almost visualizing the muscle, and watching it release*”—*C9*.

2.2.6 Breathwork: Four contributors reported using a form of breathwork alongside their psychedelic use.

“*When I'm breathing, I can feel my whole body is vibrating everywhere except for that one spot… when it lets go, it releases any kind of pain that I have*”—*C2*.

2.3 Enduring Change: All contributors perceived lasting improvements in their overall quality of life. Most contributors also felt reduced pain (9) beyond the acute psychedelic experience, and persistent increases in embodiment (9), both contributing toward effective pain management and overall comfort.
2.3.1 Pain reduction: The majority of contributors (9) experienced a lasting pain reduction, though the length of this varied considerably.

“*Every time that I have taken LSD, I have experienced relief the next day. Every single time… it helps with my state of mind the following days, but the pain specifically, I feel it always. I always get some relief. Always*”—*C8*.

“*I was waiting for (the neuralgia) to show up and it didn't. A month later, I literally fell flat on my face and had a concussion, but I did not have the neuralgia… Any kind of small accident that could affect this sphere would usually trigger the [chronic] pain and here I had a major accident and no [chronic] pain… Having a concussion and not having 3 months of neuralgia afterwards was unthinkable*”—*C5*.

“*I think the (hydromorphone) was the most effective painkiller, but even when I took that, I remember it hurting a lot the next day, and needing another one just to get out of bed. It was a sacrifice. I equate (hydromorphone) to putting your phone on vibrate—it's still ringing, it doesn't mean it's off. But the mushrooms turned the phone off. It's not masking my pain in any way. It took (the pain) out back and shot it, and I'm good now*”—*C7*.

2.3.2 Quality of Life: All contributors claimed improvements in their quality of life. This presented itself through increased function (5), independence (1), energy (5), and ability to move (4). Two contributors also reported perceived enhancements in cognitive performance (2). This contrasted with the extent of disability initially claimed and affected contributors' overall perceived wellbeing.

“*I am able to sleep, move, cook, use the bathroom, etc. I have regained mobility I have not had for years and am still in complete disbelief… I've had full mobility… I felt unusually fine ever since… It feels like my body time-machine-reverted all the way back to (when I was healthy) … I thought, maybe I can have a life again, and I laid there for maybe an hour just thinking about all the stuff I could potentially be able to do again*”—*C7*.

“*It's made me more creative and able to respond and get out of my comfort zone… I've been able to read two books a week, so I definitely feel like it's boosted my brain*”—*C5*.

2.3.3 Embodiment: The psychological construct of embodiment highlights mind-body connection and suggests subjective phenomena including feelings and behaviors are foundationally somatically informed (89). Here, the term specifically refers to this perceived connection. Both acute and enduring increases in embodiment were felt by nine contributors, departing from the dissociation and avoidance previously claimed (3). Acutely, contributors noticed this through intentional focus (4), newfound ability to move (5), and internal awareness (3). Enduring changes manifested as feelings of physical presence (7) and internal awareness (3). One person reported somatic discharge during the process of integration.

“*(I am) becoming present to my body, becoming present to the moment… becoming more aware of the relationship between how I feel and the pain that I feel*”—*C6*.

“*I'd say that the way I could feel my body was heightened. It was the presence in my body. I feel like these drugs put me in my body. I think the only way you can feel your body is when you're in it. Then you actually can feel your pain and then release it, rather than being out of the body. I think they've made me more present and in being present I was able to pinpoint exactly where the pain is*”—*C9*.

“*(I felt) the tissue memory percolating out. It really allowed this mobilization of trapped memories, the tissue memory of these trapped experiences to come out, but it did not come out during the trip, it came out as part of the processing and integration afterwards*”—*C5*.

**Table 3:** Summary of categories, themes, sub-themes, and frequencies.

**Category**	**Theme**	**Sub-theme**	**Frequency**	**%**
**POSITIVE REFRAMING**				
	***Pre-self-medication***			
	***Pain debilitation***			
	Depression		11	100%
	Interference with lives		11	100%
		Career	7	64%
		Relationships	3	27%
		Psychological disposition	10	91%
	Loss of identity		7	64%
	Desire for escape		3	27%
	Self-punishing		1	9%
	***Enduring change***			
	Mental wellbeing		11	100%
	Positive reframing		11	100%
		Acceptance	7	64%
		Agency	7	64%
		Committed action	4	36%
		Compassion	8	73%
		Hope	4	36%
		Empowerment	7	64%
	Connection		11	100%
		Self	11	100%
		Others	8	100%
		Nature	4	73%
		Spirituality	4	36%
**SOMATIC PRESENCE**				
	***Pain disability***			
	Severe pain		11	100%
	Physical impairment		11	100%
		Sleep	7	64%
		Function	11	100%
		Failure of previous treatments	7	64%
	***Acute***			
	Acutely forgetting pain		9	82%
	Feeling of health		11	100%
	Pain amplification		3	27%
	Physical release		11	100%
		Somatic discharge	2	18%
	Working with the body		8	73%
	Breathwork		4	36%
	***Enduring change***			
	Pain reduction		9	82%
	Embodiment		9	82%
	Quality of life		11	100%

## Discussion

This project was undertaken as a PPI venture for an upcoming trial, aimed at learning from the lived experiences of people with chronic pain and leveraging these data toward the development of trial design and procedures. To our knowledge, this is the first work detailing PPI for chronic pain in the context of psychedelics. Using thematic analysis, we highlight some common factors that contributors referred to as being linked to perceived successful outcomes from their self-medication. These were: positively reframed perspectives and strengthened sense of embodiment, as well as incorporating adjunct approaches. In interpreting these results however, we recognize that this work is subjective in two respects: (i) qualitative analysis is inherently subjective (90) which (ii) rests upon the subjective reports of, in this case, highly motivated contributors.

Broadly, the following are recommendations that can be taken forward from these reports into the development of the upcoming trial. Adequate preparation was highlighted as essential and included understanding the potential intensity of the experience, both physically and emotionally. All contributors in some way underscored the importance of trust, surrender, or openness for a beneficial outcome [see (91, 92)]. This included openness to the experience itself and contributors especially emphasized developing rapport and trust with their “guides” if used clinically (93–97). Using additional modalities (see below) was also viewed as a key component of the experience. Although the specific choice(s) varied between each contributor, intentionally engaging with the body through movement and/or breath was frequently highlighted. Physical catharsis, whether through physical movement, somatic discharge (see below), or even through art was also valued. This will be incorporated into the trial (details below). Finally, integration and aftercare, while not reported by everyone, were viewed as essential to the perceived longevity of therapeutic benefit.

A common psychological component seen in those with chronic pain is pain catastrophising (98). Core aspects of this phenomenon were widely expressed by contributors and include a tendency toward pessimism, helplessness, and fear (99), the result being exaggerated experience of overall discomfort. Individuals with chronic pain judge the potential threat of pain through magnification and rumination, eventually compromising their perceived agency over their situation (100). Evidence supports a causal relationship between catastrophising and pain, to varying degrees (101–105). There is also a known relationship between catastrophising and depression (104, 106) and tolerance of uncertainty (107, 108), and negative mental affect has widely been linked to worsened pain outcomes (25, 106). There can develop a self-potentiating or self-fulfilling cycle, in which the continuing experience of pain seemingly verifies the negative expectations that contribute to its maintenance. This is diametrically opposed to the tenets of optimism (109), hope (110), and openness (111) that characterize mental wellbeing, as well as improved core pain outcomes (112–114). This trend, as well as the above recommendations concerning rapport, informed our preparation process which will now include added time and additional resources.

In addition, many contributors suggested some degree of cognitive reframing, with perceived psychological states shifting from overwhelming depression, disempowerment, anxiety, and hopelessness, toward connection, acceptance, agency, and hope. This transition from catastrophising toward clarity, and in most cases, perceived optimism, reportedly ameliorated contributors' perceived ability to effectively manage their pain; they described feeling more “prepared” and no longer overwhelmed. These results speak toward cognitive, specifically positive, reframing as a driving mechanism of outcomes following psychedelic self-medication for chronic pain and bear relevance to previously discussed change mechanisms in the context of psilocybin therapy for depression research (86). Even though many contributors reported still experiencing chronic pain following their experiences, they claimed feeling a newfound sense of agency and optimism regarding their pain. The perceived relevance of these trends further put into question the mind-body dichotomy of current pain treatments, where psychological interventions are largely considered as secondary, if at all.

Changes in perspective described by contributors were often galvanized during the acute psychedelic experience, and often persisted for 2+ months. This is consistent with existing psychedelic data suggesting transdiagnostically significant long-term outcomes in openness (115), attitude (116), personality (117), depression and anxiety (86, 118) reported for months to years (119) following treatment. PPI discussions did not include assessment of specific personality traits. Therefore, no generalizations linking specific personality or psychological traits to outcomes can be made. The acute psychedelic experiences conformed to previous findings (86) and generally centered around feelings of connection, love, compassion, and trust. As with previous studies (86, 87), these sentiments, particularly connection with the self and body, endured and precipitate the development of larger shifts in perspectives. Feelings of compassion, empowerment, and hope were closely linked and contributed to a growing sense of agency and control over their pain, and life more generally. Increased subjective acceptance of pain was also claimed, specifically shifting from a defeated resignation to an active acceptance and served as an impetus to impact other aspects of life. A noteworthy minority of contributors (5) were reportedly more committed to taking an active stance in their recovery in various ways, from seeking professional help to cultivating new behaviors conducive to general health and wellbeing [see (120)]. Overall, the subjective reclamation of identity, agency, and hope emerged as drivers in the perceived efficacy of pain management. Fostering this process has consequently developed into a central aspect of our therapeutic approach spanning the duration of the study. Reinforced integration support will aim to support the longevity of changes by providing tools for independent support and additional follow-ups.

Such outcomes are the goal of numerous treatment strategies, particularly PMPs using CBT and ACT, which aim to reframe thought processes and encourage independence. While fairly widespread, PMPs are moderately effective at best, particularly at long-term follow-ups (39–42, 121). In contrast, contributors perceived a “profound shift” in perspective, which they felt contributed to the longevity of outcomes. Such claims of insight are well-supported in the psychedelic field, particularly in the context of psychological flexibility (122, 123) and further, relaxed beliefs (124) and may account for the effects reported here. Psychological flexibility facilitates well-adjusted individuals to adapt their mindset to life's ever-evolving situations, regardless of social, emotional, or stress factors (125). Existing literature points toward prodigious upregulations of neural plasticity following psychedelic experiences, translated experientially to changed perspectives (124). This is particularly relevant in populations with maladaptive, entrenched thought patterns such as depression (126), and indeed chronic pain (99). Consistent with this PPI project, therapeutically useful changes in outlook are linked to certain noetic experiences (127), allowing individuals to move “above” themselves toward a more equanimous state free of unwarranted pessimism. The cumulative results are an amalgam of self-confidence, inspiration, and acceptance (123) and directly counter the effects of pain catastrophisation.

Contributors' physical experiences of their body and pain were another point often referred to. Initially, contributors reported severe day-to-day pain, described as “burning,” “exhausting,” “looming,” “tight,” “stabbing,” and “blinding,” which left all contributors with impaired general function. Therefore, contributors were “shocked” and “in disbelief” at the perceived efficacy; 9 out of 11 contributors claimed partial or complete analgesia in the acute psychedelic stage [see (128) for similar findings]. Most contributors regained function, e.g., the ability to walk unencumbered, and described experiencing their body as “healthy.” This was reported with both high (macro) and microdoses. Effect duration varied and ranged from no relief beyond the acute experience to over a year—most contributors (8), regardless of dose taken, reported feeling reductions in pain for at least 3–5 days following their experience, with pain levels gradually increasing over time. Other changes were longer lasting though; most contributors (9) reported enduring increases in positive embodiment and interoception, which inspired a desire to treat the body better in the future.

The process toward pain relief often involves an improved sense of Somatic Presence, or “mindfulness of body.” Theories exist about the impact of trauma on somatic symptoms, e.g., chronic fatigue and chronic pain (129, 130). According to these theories, an act of intense physical expression/release, such as through shaking, vocalizing, or unconscious movement (also termed “somatic discharge”) can be profoundly cathartic and processing/working through associated symptoms (88, 131). Preliminary evidence suggests that somatic discharge may be effective not only for trauma therapy (132), but also in the context of chronic pain (32). Something akin to somatic discharge was described by two contributors in our group, who were interestingly also the only contributors who reported total analgesia indefinitely after their experience. The remaining contributors reported more subdued physical release, often associated with self-imposed elements of body- and breathwork. This largely resulted in muscular relaxation translating to subjective pain relief and heightened embodiment. These reports inspired the inclusion of behavioral investigations on interoception and body awareness in future studies Further, optional movement elements aimed at an embodied experience may be incorporated in dosing and integration sessions. Finally, references to potential elements of somatic discharge stimulated the development and implementation of a physical catharsis measure.

Importantly, not all contributors reported acute pain relief; three contributors reported amplified pain during the acute psychedelic experience. Pain reportedly grew in intensity and became “distressing” and “overpowering” before returning to baseline after the experience. While unpleasant, two contributors reported that this was a useful learning experience for them. One contributor branded this experience an outlier attributed to a “recklessly” high dose taken “rashly” and without proper consideration regarding attitude and intention. Other contributors echoed that taking very high doses in sub-optimal contexts (inadequate preparation, negative mental state, stressful environment) contributed to unfavorable experiences, although this did not necessary result in perceived pain amplification. We have therefore developed preparation sessions specifically tailored for chronic pain populations and will emphasize trust, transparency, and intention.

Due to the clandestine nature of illicit substance use, there is no standard protocol for self-medication. Interestingly, many contributors intentionally recreated a clinical setting in an effort to facilitate a therapeutic experience; they set specific intentions, laid back comfortably with their eyes closed, listened to especially chosen music, and focused on the internal journey, though this was largely without an accompanying “guide” or “sitter.”

Psychedelics were routinely combined with other adjunct practices. Supplementary modalities included breathwork, mindfulness, meditation, and movement, e.g., Yoga, Qi Gong, physical therapy exercises, and using expressive outlets such as art, journaling, and dance to manifest and process emotional content during and after the experience. These suggestions were incorporated into our therapeutic model with particular emphasis on mindfulness and movement.

The goal of this PPI project was not to produce formal research; it did not test hypotheses or draw any confirmatory claims. However, the most pertinent limitation of this project concerns both contributor and investigator bias. We invited collaborators through targeted advertisements which disproportionally attract people with positive experiences, causing selection bias. This was reinforced by lack of financial compensation and the time commitment of the project, ultimately resulting in a small, self-selected, and highly motivated cohort that was almost definitely biased by compelling personal outcomes. Further, recency, recall, response, and confirmation bias may have affected perceived efficacy and accuracy of reports. While we attempted to minimize researcher bias during analysis by cross-referencing for inter-coder-reliability, the inherently subjective process cannot eliminate potential confirmation bias. Equally, though discussions followed a consistent structure, unintentional interviewer bias (e.g., *via* body language) cannot be excluded. We reiterate therefore, that these reports are best viewed as a collection of discussions used to inform our upcoming trial and do not imply causation, generalizability of results, viability of treatment, or future study outcomes.

Beyond bias, most contributors approximated doses, challenging the accuracy of self-report quantities. Even if contributors accurately reported, there are distribution inconsistencies in both organic and synthetic materials. Additionally, evaluating the differences between microdosing and macrodosing is difficult due to potentially discrete processes (133). Duration between self-medication and discussion necessarily varied, contributing to differences in follow-up times reported and the perceived duration and efficacy of therapeutic effects. Finally, psychedelics were used in concert with other modalities, which likely confounded outcomes. If undertaken as formal research, future improvements may include more rigorous contributor selection criteria, further standardized discussion formats, and including additional cross-referencing techniques to address subjectivity concerns.

## Conclusion

In conclusion, this qualitative exploration of 11 open-ended discussions addressed perceived effects of psychedelic self-medication on aspects of chronic pain. Analysis suggested two possible processes at play during psychedelic self-medication for chronic pain: (1) Positive Reframing of contributors' relationships with their chronic pain toward perspectives of hope, empowerment, and optimism. (2) Somatic Presence fostered increased embodiment and was associated with lasting analgesia. Psychedelics were not used in isolation and were regularly combined with various other modalities including meditation, breathwork, and movement, which contributors felt impacted the success of their self-medication. Information concerning processes and complementary modalities provided useful additions to our protocol and should be considered when designing future trials.

## Data Availability Statement

The datasets presented in this article are not readily available due to their personally identifying nature. However, excerpts are available in this article. Requests to access the datasets should be directed to j.bornemann19@imperial.ac.uk.

## Ethics Statement

The Imperial Research Governance and Integrity Team (RGIT) was informed of all planned activities of the following project involving human PPI contributors. This project was undertaken as PPI and RGIT confirmed that ethical approval was not required. During conversations, PPI contributors provided verbal informed consent to participate in this study.

## Author Contributions

This project was led by JB who conducted, transcribed, and analyzed discussions. The thematic analysis was cross-referenced for inter-coder reliability by JC. LR supervised the work from its inception and advised on methodology and analysis. The manuscript was written by JB and edited by LR, MS, JC, and RC-H. All authors contributed to the article and approved the submitted version.

## Funding

This project was supported by the Centre for Psychedelic Research at Imperial College London.

## Conflict of Interest

The authors declare that the research was conducted in the absence of any commercial or financial relationships that could be construed as a potential conflict of interest.

## Publisher's Note

All claims expressed in this article are solely those of the authors and do not necessarily represent those of their affiliated organizations, or those of the publisher, the editors and the reviewers. Any product that may be evaluated in this article, or claim that may be made by its manufacturer, is not guaranteed or endorsed by the publisher.

## References

[1] Crocker JC Boylan A-M Bostock J Locock L. Is it worth it? Patient and public views on the impact of their involvement in health research and its assessment: a UK-based qualitative interview study. Health Expect. (2016) 20:519–28. 10.1111/hex.12479 27338242 PMC5433537

[2] National Institute for Health Research: Research Design Service South Central. (n.d.). Patient and Public Involvement (PPI). Available online at: https://www.rds-sc.nihr.ac.uk/ppi-information-resources/ (accessed June 9, 2021).

[3] Ennis L Wykes T. Impact of patient involvement in mental health research: longitudinal study. Br J Psychiatry. (2013) 203:381–6. 10.1192/bjp.bp.112.119818 24029538

[4] Brett J Staniszewska S Mockford C Herron-Marx S Hughes J Tysall C. A systematic review of the impact of Patient and Public Involvement on service users, researchers and communities. Patient. (2014) 7:387–95. 10.1007/s40271-014-0065-0 25034612

[5] Close JB Bornemann J Piggin M Jayacodi S Luan LX Carhart-Harris R. A Strategy for Patient and Public Involvement in Psychedelic Research *Front*. Psychiatry. (2021). 12:1696 10.3389/fpsyt.2021.727496 34658961 PMC8514741

[6] Troya MI Bartlam B Chew-Graham C. Involving the public in health research in Latin America: making the case for mental health. Rev Panam Salud Públ. (2018) 42:1–6. 10.26633/RPSP.2018.45 31093073 PMC6386097

[7] National Institute for Health Research. Public Involvement in Research: Values and Principles Framework. Involve (2015). Available online at: https://www.invo.org.uk/wp-content/uploads/2017/08/Values-Principles-framework-Jan2016.pdf (accessed October, 2015).

[8] Wernicke S de Witt Huberts J Wippert P-M. The pain of being misunderstood: invalidation of pain complaints in chronic low back pain patients. J Health Psychol. (2016) 22:135–47. 10.1177/1359105315596371 26276505

[9] Kool MB van Middendorp H Lumley MA Bijlsma JWJ Geenen R. Social support and invalidation by others contribute uniquely to the understanding of physical and mental health of patients with rheumatic diseases. J Health Psychol. (2013) 18:86–95. 10.1177/1359105312436438 22363049

[10] Boote J Baird W Beecroft C. Public involvement at the design stage of primary health research: a narrative review of case examples. Health Policy. (2010) 95:10–23. 10.1016/j.healthpol.2009.11.007 19963299

[11] Engage. (n.d.). One to One Interview - Personal and Public Involvement (PPI). Available online at: http://engage.hscni.net/get-involved/involving-people/methods-of-involvement/interviews/ (accessed June 15, 2021).

[12] Tomlinson J Medlinskiene K Cheong V-L Khan S Fylan B. Patient and Public Involvement in designing and conducting doctoral research: the whys and the hows. Res Involv Engag. (2019) 5:23. 10.1186/s40900-019-0155-1 31428458 PMC6697942

[13] Harrison M Palmer R. Exploring Patient and Public Involvement in stroke research: a qualitative study. Disabil Rehabil. (2015) 37:2174–83. 10.3109/09638288.2014.1001525 25598139

[14] Dudley L Gamble C Preston J Buck D Hanley B Williamson P. What difference does Patient and Public Involvement make and what are its pathways to impact? Qualitative study of patients and researchers from a cohort of randomised clinical trials. PLoS One. (2015) 10:e0128817. 10.1371/journal.pone.0128817 26053063 PMC4459695

[15] Ali K Roffe C Crome P. What patients want: consumer involvement in the design of a randomized controlled trial of routine oxygen supplementation after acute stroke. Stroke. (2006) 37:865–71. 10.1161/01.STR.0000204053.36966.80 16456122

[16] Burke A Mathias J Denson L. Psychological functioning of people living with chronic pain: a meta-analytic review. Br J Clin Psychol. (2015) 54:345–60. 10.1111/bjc.12078 25772553

[17] Vos T. Global, regional, and national incidence, prevalence, and years lived with disability for 328 diseases and injuries for 195 countries, 1990-2016: a systematic analysis for the Global Burden of Disease Study 2016. Lancet. (2017) 390:1211–59. 10.1016/S0140-6736(17)32154-2 28919117 PMC5605509

[18] www.iasp-pain.org. IASP Task Force for the Classification of Chronic Pain in ICD-11 Prepares New Criteria on Postsurgical Posttraumatic Pain. IASP (2016). Available online at: https://www.iasp-pain.org/PublicationsNews/NewsDetail.aspx?ItemNumber=5134#:~:text=For%20the%20purpose%20of%20the (accessed June 9, 2021).

[19] Treede R-D Rief W Barke A Aziz Q Bennett MI Benoliel R. A classification of chronic pain for ICD-11. Pain. (2015) 156:1003–7. 10.1097/j.pain.0000000000000160 25844555 PMC4450869

[20] Phillips C. The cost and burden of chronic pain. Rev Pain. (2009) 3:2–5. 10.1177/204946370900300102 26526940 PMC4590036

[21] Duenas M Ojeda B Salazar A Mico J Failde I. A review of chronic pain impact on patients, their social environment and the health care system. J Pain Res. (2016) 9:457–67. 10.2147/JPR.S105892 27418853 PMC4935027

[22] Dopson L. Role of pain management programmes in chronic pain. Nurs Stand. (2010) 25:35–40. 10.7748/ns2010.12.25.13.35.c8120 21207824

[23] Tsang A Von Korff M Lee S Alonso J Karam E Angermeyer MC. Common chronic pain conditions in developed and developing countries: gender and age differences and comorbidity with depression-anxiety disorders. J Pain. (2008) 9:883–9. 10.1016/j.jpain.2008.05.005 18602869

[24] Feizerfan A Sheh G. Transition from acute to chronic pain. Contin Educ Anaesth Crit Care Pain. (2015) 15:98–102. 10.1093/bjaceaccp/mku044

[25] Sheng J Liu S Wang Y Cui R Zhang X. The link between depression and chronic pain: neural mechanisms in the brain. Neural Plast. (2017) 2017:9724371. 10.1155/2017/9724371 28706741 PMC5494581

[26] Anekar AA Cascella M. WHO Analgesic Ladder. (2020). Available online at: https://www.ncbi.nlm.nih.gov/books/NBK554435/ (accessed May 18, 2021).

[27] www.nice.org.uk. Overview | Chronic Pain (Primary Secondary) in Over 16s: Assessment of All Chronic Pain Management of Chronic Primary Pain | Guidance. NICE (2021). Available online at: https://www.nice.org.uk/guidance/NG193 (accessed April 7, 2021).

[28] Cohen SP Vase L Hooten WM. Chronic pain: an update on burden, best practices, and new advances. Lancet. (2021) 397:2082–97. 10.1016/S0140-6736(21)00393-7 34062143

[29] Coussens NP Sittampalam GS Jonson SG Hall MD Gorby HE Tamiz AP. The opioid crisis and the future of addiction and pain therapeutics. J Pharmacol Exp Ther. (2019) 371:396–408. 10.1124/jpet.119.259408 31481516 PMC6863454

[30] Orhan ME Bilgin F Ergin A Dere K Güzeldemir ME. Pain treatment practice according to the WHO analgesic ladder in cancer patients: eight years' experience of a single center]. Agri. (2008) 20:37–43. 19117155

[31] Alford DP German JS Samet JH Cheng DM Lloyd-Travaglini CA Saitz R. Primary care patients with drug use report chronic pain and self-medicate with alcohol and other drugs. J Gen Intern Med. (2016) 31:486–91. 10.1007/s11606-016-3586-5 26809204 PMC4835374

[32] Andersen TE Lahav Y Ellegaard H Manniche C. A randomized controlled trial of brief Somatic Experiencing for chronic low back pain and comorbid post-traumatic stress disorder symptoms. Eur J Psychotraumatol. (2017) 8:1331108. 10.1080/20008198.2017.1331108 28680540 PMC5489867

[33] Hofmann SG Asnaani A Vonk IJJ Sawyer AT Fang A. The efficacy of cognitive behavioral therapy: a review of meta-analyses. Cogn Ther Res. (2012) 36:427–40. 10.1007/s10608-012-9476-1 23459093 PMC3584580

[34] Jiménez F. Ruiz. Acceptance and commitment therapy versus traditional cognitive behavioral therapy: a systematic review and meta-analysis of current empirical evidence. Int J Psychol Psychol Ther. (2012) 12:333–58.

[35] Mueser KT Gottlieb JD Xie H Lu W Yanos PT Rosenberg SD. Evaluation of cognitive restructuring for post-traumatic stress disorder in people with severe mental illness. Br J Psychiatry. (2015) 206:501–8. 10.1192/bjp.bp.114.147926 25858178 PMC4450219

[36] Larsson A Hooper N Osborne LA Bennett P McHugh L. Using brief cognitive restructuring and cognitive defusion techniques to cope with negative thoughts. Behav Modif. (2015) 40:452–82. 10.1177/0145445515621488 26685210

[37] Lambert NM Fincham FD Stillman TF. Gratitude and depressive symptoms: the role of positive reframing and positive emotion. Cogn Emot. (2012) 26:615–33. 10.1080/02699931.2011.595393 21923564

[38] Finan PH Garland EL. The role of positive affect in pain and its treatment. Clin J Pain. (2015) 31:177–87. 10.1097/AJP.0000000000000092 24751543 PMC4201897

[39] Smith JG Knight L Stewart A Smith EL McCracken LM. Clinical effectiveness of a residential pain management programme - comparing a large recent sample with previously published outcome data. Br J Pain. (2016) 10:46–58. 10.1177/2049463715601445 27551411 PMC4977965

[40] Hughes LS Clark J Colclough JA Dale E McMillan D. Acceptance and Commitment Therapy (ACT) for chronic pain. Clin J Pain. (2017) 33:552–68. 10.1097/AJP.0000000000000425 27479642

[41] Wilson IR. Management of chronic pain through pain management programmes. Br Med Bull. (2017) 124:1–10. 10.1093/bmb/ldx032 28927228

[42] Schütze R Rees C Smith A Slater H Campbell JM O'Sullivan P. How can we best reduce pain catastrophizing in adults with chronic noncancer pain? A systematic review and meta-analysis. J Pain. (2018) 19:233–56. 10.1016/j.jpain.2017.09.010 29122652

[43] Dysvik E Kvaløy JT Natvig GK. The effectiveness of an improved multidisciplinary pain management programme: a 6- and 12-month follow-up study. J Adv Nurs. (2011) 68:1061–72. 10.1111/j.1365-2648.2011.05810.x 22050304

[44] Burke D Lennon O Blake C Nolan M Barry S Smith E. An internet-delivered cognitive behavioural therapy pain management programme for spinal cord injury pain: a randomized controlled trial. Eur J Pain. (2019) 23:1264–82. 10.1002/ejp.1402 31002442

[45] Osório FdL Sanches RF Macedo LR dos Santos RG Maia-de-Oliveira JP. Antidepressant effects of a single dose of ayahuasca in patients with recurrent depression: a preliminary report. Rev Bras Psiquiatr. (2015) 37:13–20. 10.1590/1516-4446-2014-1496 25806551

[46] Palhano-Fontes F Barreto D Onias H Andrade KC Novaes MM Pessoa JA. Rapid antidepressant effects of the psychedelic ayahuasca in treatment-resistant depression: a randomized placebo-controlled trial. Psychol Med. (2018) 49:655–63. 10.1017/S0033291718001356 29903051 PMC6378413

[47] Carhart-Harris R Giribaldi B Watts R Baker-Jones M Murphy-Beiner A Murphy R. Trial of psilocybin versus escitalopram for depression. N Engl J Med. (2021) 384:1402–11. 10.1056/NEJMoa2032994 33852780

[48] Bogenschutz MP Forcehimes AA Pommy JA Wilcox CE Barbosa PCR Strassman RJ. Psilocybin-assisted treatment for alcohol dependence: a proof-of-concept study. J Psychopharmacol. (2015) 29:289–99. 10.1177/0269881114565144 25586396

[49] Johnson MW Garcia-Romeu A Griffiths RR. Long-term follow-up of psilocybin-facilitated smoking cessation. Am J Drug Alcohol Abuse. (2016) 43:55–60. 10.3109/00952990.2016.1170135 27441452 PMC5641975

[50] Grob CS Danforth AL Chopra GS Hagerty M McKay CR Halberstadt AL. Pilot study of psilocybin treatment for anxiety in patients with advanced-stage cancer. Arch Gen Psychiatry. (2011) 68:71. 10.1001/archgenpsychiatry.2010.116 20819978

[51] Gasser P Holstein D Michel Y Doblin R Yazar-Klosinski B Passie T. Safety and efficacy of lysergic acid diethylamide-assisted psychotherapy for anxiety associated with life-threatening diseases. J Nerv Ment Dis. (2014) 202:513–20. 10.1097/NMD.0000000000000113 24594678 PMC4086777

[52] Griffiths RR Johnson MW Carducci MA Umbricht A Richards WA Richards BD. Psilocybin produces substantial and sustained decreases in depression and anxiety in patients with life-threatening cancer: a randomized double-blind trial. J Psychopharmacol. (2016) 30:1181–97. 10.1177/0269881116675513 27909165 PMC5367557

[53] Ross S Bossis A Guss J Agin-Liebes G Malone T Cohen B. Rapid and sustained symptom reduction following psilocybin treatment for anxiety and depression in patients with life-threatening cancer: a randomized controlled trial. J Psychopharmacol. (2016) 30:1165–80. 10.1177/0269881116675512 27909164 PMC5367551

[54] Nichols DE. Psychedelics. Pharmacol Rev. (2016) 68:264–355. 10.1124/pr.115.011478 26841800 PMC4813425

[55] Barrett FS Bradstreet MP Leoutsakos J-MS Johnson MW Griffiths RR. The Challenging Experience Questionnaire: Characterization of challenging experiences with psilocybin mushrooms. J Psychopharmacol. (2016) 30:1279–95. 10.1177/0269881116678781 27856683 PMC5549781

[56] Kast E. LSD used as analgesic. JAMA. (1964) 187:A33. 10.1001/jama.1964.03060140099054

[57] Kast E. LSD and the Dying Patient. Chic Med Sch Q. (1966) 26:80–7. 4163076

[58] Kast E. Attenuation of anticipation: a therapeutic use of lysergic acid diethylamide. Psychiatr Q. (1967) 41:646–57. 10.1007/BF01575629 4169685

[59] Kuromaru S Okada S Hanada M Kasahara Y Sakamoto K. The effect of LSD on the phantom limb phenomenon. Lancet. (1967) 87:22–7. 6043203

[60] Pahnke W Kurland A Goodman L Richards W. LSD-assisted psychotherapy with terminal cancer patients. Curr Psychiatr Ther. (1969) 9:144–52. 5348915

[61] Grof S Goodman L Richards W Kurland A. LSD-Assisted psychotherapy in patients with terminal cancer. Int Pharmacopsychiatry. (1973) 8:129–44. 10.1159/000467984 4140164

[62] Sewell R Halpern J Pope H. Response of cluster headache to psilocybin and LSD. Neurology. (2006) 66:1920–2. 10.1212/01.wnl.0000219761.05466.43 16801660

[63] Karst M Halpern J Bernateck M Passie T. The non-hallucinogen 2-bromo-lysergic acid diethylamide as preventative treatment for cluster headache: an open, non-randomized case series. Cephalalgia. (2010) 30:1140–4. 10.1177/0333102410363490 20713566

[64] Schindler E Gottschalk C Weil M Shapiro R Wright D Sewell R. Indoleamine hallucinogens in cluster headache: results of the clusterbusters medication use survey. J Psychoactive Drugs. (2015) 47:372–81. 10.1080/02791072.2015.1107664 26595349

[65] Whelan A Johnson M. Lysergic acid diethylamide and psilocybin for the management of patients with persistent pain: a potentia role? Pain Manag. (2018) 8:217–29. 10.2217/pmt-2017-0068 29722608

[66] Castellanos J Woolley C Bruno K Zeidan F Halberstadt A Furnish T. Chronic pain and psychedelics: a review and proposed mechanism of action. Region Anesth Pain Med. (2020) 45:486–94. 10.1136/rapm-2020-101273 32371500

[67] Bardin L. The complex role of serotonin and 5-HT receptors in chronic pain. Behav Pharmacol. (2011) 22:390–404. 10.1097/FBP.0b013e328349aae4 21808193

[68] Nau F Yu B Martin D Nichols C. Serotonin 5-HT2A receptor activation blocks TNF-α mediated inflammation *in vivo*. PLoS One. (2013) 8:e75426. 10.1371/journal.pone.0075426 24098382 PMC3788795

[69] Flanagan T Nichols C. Psychedelics as anti-inflammatory agents. Int Rev Psychiatry. (2018) 30:363–75. 10.1080/09540261.2018.1481827 30102081

[70] Nardai S László M Szabó A Alpár A Hanics J Zahola P. N,N-dimethyltryptamine reduces infarct size and improves functional recovery following transient focal brain ischemia in rats. Exp Neurol. (2020) 327:113245. 10.1016/j.expneurol.2020.113245 32067950

[71] Kozłowska U Klimczak A Wiatr K Figiel M. The DMT and psilocin treatment changes CD11b+ activated microglia immunological phenotype. BioRXiv. (2021). 10.1101/2021.03.07.434103

[72] Szabo A. Psychedelics and immunomodulation: novel approaches and therapeutic opportunities. Front Immunol. (2015) 6:358. 10.3389/fimmu.2015.00358 26236313 PMC4500993

[73] Kast EC Collins VJ. Study of lysergic acid diethylamide as an analgesic agent. Anesth Analg. (1964) 43:285–91. 10.1213/00000539-196405000-00013 14169837

[74] Pestana J Beccaria F Petrilli E. Psychedelic substance use in the Reddit psychonaut community. A qualitative study on motives and modalities. Drugs Alcohol Today. (2020). 10.1108/DAT-03-2020-0016

[75] www.samhsa.gov. (n.d.). Section 7 PE Tables - Results from the 2019 National Survey on Drug Use and Health: Detailed Tables, SAMHSA, CBHSQ. Available online at: https://www.samhsa.gov/data/sites/default/files/reports/rpt29394/NSDUHDetailedTabs2019/NSDUHDetTabsSect7pe2019.htm#tab7-40a (accessed June 9, 2021).

[76] Mason NL Kuypers KPC. Mental health of a self-selected sample of psychedelic users and self-medication practices with psychedelics. J Psychedelic Stud. (2018) 2:45–52. 10.1556/2054.2018.006

[77] www.erowid.org. (n.d.). Mushrooms - Erowid Exp – “Anecdotal Cure for Back Pain?” Available online at: https://www.erowid.org/experiences/exp.php?ID=65650 (accessed May 5, 2021).

[78] www.erowid.org. (n.d.). Mushrooms - P. cubensis and Vitamins/Supplements - Erowid Exp – “For the Pain of Fbromyalgia.” Available online at: https://www.erowid.org/experiences/exp.php?ID=68760 (accessed May 5, 2021).

[79] Reddit/Ehlers Danlos. (n.d.). Experiences With Psychedelics. Available online at: https://www.reddit.com/r/ehlersdanlos/comments/atrmqv/experiences_with_psychedelics (accessed February 23, 2019).

[80] Andersson M Persson M Kjellgren A. Psychoactive substances as a last resort-a qualitative study of self-treatment of migraine and cluster headaches. Harm Reduct J. (2017) 14:60. 10.1186/s12954-017-0186-6 28870224 PMC5584001

[81] Clusterbusters. (n.d.). Clusterbusters. Available online at: https://clusterbusters.org/ (accessed May 5, 2021).

[82] Hawker GA Mian S Kendzerska T French M. Measures of adult pain: Visual Analog Scale for Pain (VAS Pain), Numeric Rating Scale for Pain (NRS Pain), McGill Pain Questionnaire (MPQ), Short-Form McGill Pain Questionnaire (SF-MPQ), Chronic Pain Grade Scale (CPGS), Short Form-36 Bodily Pain Scale (SF. Arthritis Care Res. (2011) 63:S240–S52. 10.1002/acr.20543 22588748

[83] Nowell LS Norris JM White DE Moules NJ. Thematic analysis: striving to meet the trustworthiness criteria. Int J Qual Methods. (2017) 16:1–13. 10.1177/1609406917733847

[84] Flourish. (n.d.). Flourish | Data Visualization and Storytelling. Available online at: https://flourish.studio

[85] Staniszewska S Brett J Simera I Seers K Mockford C Goodlad S. GRIPP2 reporting checklists: tools to improve reporting of Patient and Public Involvement in research. BMJ. (2017) 358:j3453. 10.1136/bmj.j3453 28768629 PMC5539518

[86] Watts R Day C Krzanowski J Nutt D Carhart-Harris R. Patients' accounts of increased “connectedness” and “acceptance” after psilocybin for treatment-resistant depression. J Human Psychol. (2017) 57:520–64. 10.1177/0022167817709585

[87] Forstmann M Yudkin DA Prosser AMB Heller SM Crockett MJ. Transformative experience and social connectedness mediate the mood-enhancing effects of psychedelic use in naturalistic settings. Proc Natl Acad Sci U S A. (2020) 117:2338–46. 10.1073/pnas.1918477117 31964815 PMC7007572

[88] Payne P Levine PA Crane-Godreau MA. Somatic experiencing: using interoception and proprioception as core elements of trauma therapy. Front Psychol. (2015) 6:93. 10.3389/fpsyg.2015.00093 25699005 PMC4316402

[89] Meier BP Schnall S Schwarz N Bargh JA. Embodiment in social psychology. Top Cogn Sci. (2012) 4:705–16. 10.1111/j.1756-8765.2012.01212.x 22777820

[90] Myers M. Qualitative research and the generalizability question: standing firm with proteus. Qual Rep. (2000) 4:9. 10.46743/2160-3715/2000.2925

[91] Haijen ECHM Kaelen M Roseman L Timmermann C Kettner H Russ S. Predicting responses to psychedelics: a prospective study. Front Pharmacol. (2018) 9:897. 10.3389/fphar.2018.00897 30450045 PMC6225734

[92] Russ SL Carhart-Harris RL Maruyama G Elliott MS. Replication and extension of a model predicting response to psilocybin. Psychopharmacology. (2019) 236:3221–30. 10.1007/s00213-019-05279-z 31203401

[93] Fadiman J. The Psychedelic Explorer's Guide: Safe, Therapeutic, and Sacred Journeys. Rochester, NY: Park Street Press (2011).

[94] Richards WA William Barnard G. Sacred Knowledge: Psychedelics and Religious Experiences. New York, NY: Columbia University Press (2015).

[95] Metzner R. Allies for Awakening: Guidelines for Productive and Safe Experiences With Entheogens. Berkeley, CA: Green Earth Foundation and Regent Press (2015).

[96] Phelps J. Training psychedelic therapists. In: Winkelman M Sessa B editors. Advances in Psychedelic Medicine: State of the Art Therapeutic Applications. Santa Barbara, USA: Praeger Books (2019). p. 274–94.

[97] Haden M. Manual for Psychedelic Guides, 2nd Edn. Vancouver, Canada: Kyandara Publishing (2020).

[98] Severeijns R Vlaeyen JW van den Hout MA Weber WE. Pain catastrophizing predicts pain intensity, disability, and psychological distress independent of the level of physical impairment. Clin J Pain. (2001) 17:165–72. 10.1097/00002508-200106000-00009 11444718

[99] Quartana PJ Campbell CM Edwards RR. Pain catastrophizing: a critical review. Expert Rev Neurother. (2009) 9:745–58. 10.1586/ern.09.34 19402782 PMC2696024

[100] Leung L. Pain catastrophizing: an updated review. Indian J Psychol Med. (2012) 34:204. 10.4103/0253-7176.106012 23441031 PMC3573569

[101] Crombez G Vlaeyen JWS Heuts PHTG Lysens R. Pain-related fear is more disabling than pain itself: evidence on the role of pain-related fear in chronic back pain disability. Pain. (1999) 80:329–39. 10.1016/S0304-3959(98)00229-2 10204746

[102] Swinkels-Meewisse IEJ Roelofs J Oostendorp RAB Verbeek ALM Vlaeyen JWS. Acute low back pain: pain-related fear and pain catastrophizing influence physical performance and perceived disability. Pain. (2006) 120:36–43. 10.1016/j.pain.2005.10.005 16359797

[103] Thibault P Loisel P Durand M-J Catchlove R Sullivan MJL. Psychological predictors of pain expression and activity intolerance in chronic pain patients. Pain. (2008) 139:47–54. 10.1016/j.pain.2008.02.029 18430518

[104] Skoufa L Givissis P Simos G. Pain catastrophizing, depression and their impact on pain intensity. Int J Novel Res Healthcare Nurs. (2015) 2:59–65.

[105] Ellingsen D-M Beissner F Moher Alsady T Lazaridou A Paschali M Berry M. A picture is worth a thousand words: linking fibromyalgia pain widespreadness from digital pain drawings with pain catastrophizing and brain cross-network connectivity. Pain. (2021) 162:1352–63. 10.1097/j.pain.0000000000002134 33230008 PMC8049950

[106] Nicholas MK Coulston CM Asghari A Malhi GS. Depressive symptoms in patients with chronic pain. Med J Aust. (2009)190:S66–S70. 10.5694/j.1326-5377.2009.tb02473.x 19351296

[107] Rondung E Ekdahl J Sundin Ö. Potential mechanisms in fear of birth: the role of pain catastrophizing and intolerance of uncertainty. Birth. (2018) 46:61–8. 10.1111/birt.12368 29954044

[108] Hahn I. Examining the Role of Intolerance of Uncertainty in the Experience of Pain. ourspace.uregina.ca (2019). Available online at: https://ourspace.uregina.ca/handle/10294/9013 (accessed June 21, 2021).

[109] Conversano C Rotondo A Lensi E Della Vista O Arpone F Reda MA. Optimism and its impact on mental and physical well-being. Clin Pract Epidemiol Ment Health. (2010) 6:25–9. 10.2174/17450179010060100025 20592964 PMC2894461

[110] Acharya T Agius M. The importance of hope against other factors in the recovery of mental illness. Psychiatr Danub. (2017) 29(Suppl. 3):619–22. 28953841

[111] Kokko K Rantanen J Pulkkinen L. Associations between mental well-being and personality from a life span perspective. In: Blatný M editors. Personality and Well-being Across the Life-Span. London: Palgrave Macmillan (2015). p. 134–59.

[112] Ramírez-Maestre C Esteve R López AE. The role of optimism and pessimism in chronic pain patients adjustment. Span J Psychol. (2012) 15:286–94. 10.5209/rev_SJOP.2012.v15.n1.37335 22379718

[113] Goodin BR Bulls HW. Optimism and the experience of pain: benefits of seeing the glass as half full. Curr Pain Headache Rep. (2013) 17:329. 10.1007/s11916-013-0329-8 23519832 PMC3935764

[114] Katsimigos A-M O'Beirne S Harmon D. Hope and chronic pain-a systematic review. Irish J Med Sci. (2020) 190:307–12. 10.1007/s11845-020-02251-1 32451764

[115] Madsen MK Fisher PM Stenbæk DS Kristiansen S Burmester D Lehel S. A single psilocybin dose is associated with long-term increased mindfulness, preceded by a proportional change in neocortical 5-HT2A receptor binding. Eur Neuropsychopharmacol. (2020) 33:71–80. 10.1016/j.euroneuro.2020.02.001 32146028

[116] Griffiths RR Johnson MW Richards WA Richards BD McCann U Jesse R. Psilocybin occasioned mystical-type experiences: immediate and persisting dose-related effects. Psychopharmacology. (2011) 218:649–65. 10.1007/s00213-011-2358-5 21674151 PMC3308357

[117] Erritzoe D Roseman L Nour MM MacLean K Kaelen M Nutt DJ. Effects of psilocybin therapy on personality structure. Acta Psychiatr Scand. (2018) 138:368–78. 10.1111/acps.12904 29923178 PMC6220878

[118] Carhart-Harris RL Bolstridge M Day CMJ Rucker J Watts R Erritzoe DE. Psilocybin with psychological support for treatment-resistant depression: six-month follow-up. Psychopharmacology. (2017) 235:399–408. 10.1007/s00213-017-4771-x 29119217 PMC5813086

[119] Agin-Liebes GI Malone T Yalch MM Mennenga SE Ponté KL Guss J. Long-term follow-up of psilocybin-assisted psychotherapy for psychiatric and existential distress in patients with life-threatening cancer. J Psychopharmacol. (2020) 34:155–66. 10.1177/0269881119897615 31916890

[120] Teixeira PJ Johnson MW Timmermann C Watts R Erritzoe D Douglass H. Psychedelics and health behaviour change. J Psychopharmacol. (2021). 10.1177/02698811211008554 34053342 PMC8801670

[121] McCracken LM Vowles KE. A prospective analysis of acceptance of pain and values-based action in patients with chronic pain. Health Psychol. (2008) 27:215–20. 10.1037/0278-6133.27.2.215 18377140

[122] Davis AK Barrett FS Griffiths RR. Psychological flexibility mediates the relations between acute psychedelic effects and subjective decreases in depression and anxiety. J Context Behav Sci. (2020) 15:39–45. 10.1016/j.jcbs.2019.11.004 32864325 PMC7451132

[123] Watts R Luoma JB. The use of the psychological flexibility model to support psychedelic assisted therapy. J Context Behav Sci. (2020) 15:92–102. 10.1016/j.jcbs.2019.12.004

[124] Carhart-Harris RL Friston KJ. REBUS and the anarchic brain: toward a unified model of the brain action of psychedelics. Pharmacol Rev. (2019) 71:316–44. 10.1124/pr.118.017160 31221820 PMC6588209

[125] Gloster AT Meyer AH Lieb R. Psychological flexibility as a malleable public health target: evidence from a representative sample. J Context Behav Sci. (2017) 6:166–71. 10.1016/j.jcbs.2017.02.003

[126] Clark DA. Cognitive restructuring. In: Stefan G. Hofmann editor. The Wiley Handbook of Cognitive Behavioral Therapy. Chichester, UK: Wiley Blackwell (2013). p. 1–22.

[127] Hayes SC Law S Malady M Zhu Z Bai X. The centrality of sense of self in psychological flexibility processes: what the neurobiological and psychological correlates of psychedelics suggest. J Context Behav Sci. (2020) 15:30–8. 10.1016/j.jcbs.2019.11.005

[128] Ramaekers JG Hutten N Mason NL Dolder P Theunissen EL Holze F. A low dose of lysergic acid diethylamide decreases pain perception in healthy volunteers. J Psychopharmacol. (2020) 35:398–405. 10.1177/0269881120940937 32842825 PMC8054163

[129] Levine PA. Waking the Tiger - Healing Trauma: The Innate Capacity to Transform Overwhelming Experiences Berkeley, CA: North Atlantic Books (1997).

[130] van der Kolk B. The Body Keeps the Score: Brain, Mind and Body in the Healing of Trauma. New York, NY: Penguin Books (2015).

[131] Totton N. Body Psychotherapy: An Introduction. Philadelphia, PA: Open University Press (2003).

[132] Brom D Stokar Y Lawi C Nuriel-Porat V Ziv Y Lerner K. Somatic experiencing for posttraumatic stress disorder: a randomized controlled outcome study. J Trauma Stress. (2017) 30:304–12. 10.1002/jts.22189 28585761 PMC5518443

[133] Anderson T Petranker R Christopher A Rosenbaum D Weissman C Dinh-Williams L-A. Psychedelic microdosing benefits and challenges: an empirical codebook. Harm Reduct J. (2019) 16:43. 10.1186/s12954-019-0308-4 31288862 PMC6617883

